# Inducing visual attention through audiovisual stimuli: Can synchronous sound be a salient event?

**DOI:** 10.1177/03010066231208127

**Published:** 2023-10-23

**Authors:** Inês Salselas, Frederico Pereira, Emanuel Sousa

**Affiliations:** Polytechnic Institute of Porto, Portugal; 202623 Centro de computação Gráfica, Portugal; 202623 Centro de computação Gráfica, Portugal; Centro Algoritmi, Portugal

**Keywords:** synchrony, audiovisual attention

## Abstract

We present an experimental research aiming to explore how spatial attention may be biased through auditory stimuli. In particular, we investigate how synchronous sound and image may affect attention and increase the saliency of the audiovisual event. We have designed and implemented an experimental study where subjects, wearing an eye-tracking system, were examined regarding their gaze toward the audiovisual stimuli being displayed. The audiovisual stimuli were specifically tailored for this experiment, consisting of videos contrasting in terms of *Synch Points* (i.e., moments where a visual event is associated with a visible trigger movement, synchronous with its correspondent sound). While consistency across audiovisual sensory modalities revealed to be an attention-drawing feature, when combined with synchrony, it clearly emphasized the biasing, triggering orienting, that is, focal attention towards the particular scene that contains the Synch Point. Consequently, results revealed synchrony to be a saliency factor, contributing to the strengthening of the focal attention.

In today's increasingly complex multimedia landscape, the interaction between auditory and visual stimuli plays a pivotal role in shaping our perception and directing our attention. Within the context of the research on multisensory attention, this study endeavors to explore the intricate dynamics of attentional allocation concerning audiovisual stimuli, specifically focusing on the impact of synchronized auditory and visual cues on capturing and directing attention.

Attention is a concept that has been ubiquitous in many fields of perception and cognition and, consequently, it has been studied from numerous perspectives. In this study, we refer to attention as the set of mechanisms involved in the selection, regulation, and maintenance of focus on the most significant information to ensure priority processing and, consequently, to prepare future behavior (Chun et al., 2011).

Attention has been regarded as a system comprising multiple components, independent but interrelated, that can be summarized into three major functions: orienting, executive control, and alerting (Petersen & Posner, 2012; Posner & Boies, 1971). Orienting is the process of moving attention towards a certain location, implying that incoming signals are amplified, eliciting the detection of a salient event. Orienting is, therefore, the subsystem of attention with particular interest for the scope of this research. By attending to a specific location through orienting, the target stimulus will be given priority for a more efficient processing (Posner & Petersen, 1990).

It is rare the occasion where our perception of the world involves exclusively hearing or only seeing (Spence, 2018). Our experience of the world is, in general, multimodal (Handel, 2006) and attention is no exception, involving the coordination of all input stimuli in a cross-modal manner (Driver & Spence, 1998; Spence, 2021; Turoman et al., 2021). Consequently, the persistent relation between attentional systems implies substantial limitations to an independent processing of information presented in both auditory and visual sensory modalities. This processing dependency points to an audiovisual integration hypothesis of attention (Andersen & Mamassian, 2008; Spence & Driver, 1997a).

When attention is captured by a salient event towards a particular location in a single sensory modality, it will likely yield slight changes of attention in other modalities. Particularly, sound can be more effective in attracting spatial attention to locations than visual signals (Spence & Driver, 1997a). Humans frequently depend on audition to direct visual attention, especially when signals are present outside the visual field. For example, unexpected sounds (the exogenous case) do not only attract auditory attention but also call for visual attention to their location (Cohen-Lhyver et al., 2020; Spence & Driver, 1997a).

In a study by Iordanescu et al. (2008), redundancy is proposed as a central factor to improve the audiovisual signal strength and reliability, given that information noise (environmental and sensory) is frequently uncorrelated across the two modalities. According to the study, redundant (consistent) information provided by both modalities facilitates object identification, that is, an auditory event matching the expectation induced by the target visual object, facilitates its identification. Similarly, redundancy provided by spatial congruency contributes to audiovisual integration, enhancing visual target detection. The perceived spatial location equivalence, between auditory and visual stimuli, enhances attentional attractiveness (Bolognini et al., 2005; Spence & Driver, 1997b) (although Noesselt et al. (2008) have shown that space concurrency is not necessarily a requirement for sounds to increase temporal visual accuracy).

Synchrony has been pointed out as a potential saliency factor in audiovisual attention, with studies suggesting that auditory stimuli are sufficient to increase visual search speed when temporal synchronous condition is assured (Van der Burg et al., 2008, 2010; Van Eijk et al., 2008). Interestingly, Van der Burg's et al. (2008) also showed that an auditory stimuli synchronous with a visual distractor can increase the time required in finding a concurrent visual target. The non-spatial auditory event guides visual attention toward the location of the visual object, in an automatic manner, if the condition of synchrony is guaranteed. This reinforces the hypothesis of a joint integration of audiovisual information that leads to the amplification of phenomenal visual saliency.

Synchrony, thus, has been observed as a crucial factor that captures and drives audiovisual attention. However, this audiovisual consistency or coherency across sensory modalities has been experimentally addressed mainly using stimuli such as warning auditory signals (*bleeps and pops*). Artificial tones are not representative of the diversity and richness of the sensory information encountered in the real world and, thus, are not considered ecological stimuli (Zacharov, 2019). For this reason, there has been a growing concern about the ecological validity of stimuli, as it is important to ensure that laboratory findings on multisensory phenomena can be generalized to real-world settings (Kvasova et al., 2019; Neisser, 1976; Osborne-Crowley, 2020; Parsons, 2015; Risko et al., 2016; Shamay-Tsoory & Mendelsohn, 2019; Soto-Faraco et al., 2019; Spence & Ho, 2015; van Eijk et al., 2008).

Furthermore, new visual orienting challenges emerge from recent technological advancements, specifically in immersive audiovisual environments (e.g., Virtual Reality, 360 videos). In non-linear media contexts, where image surrounds the user and decisions regarding whether and when to look at can be made (the user-dependent framing paradigm), different strategies are required compared to those employed in traditional storytelling (Dooley, 2017; Nielsen et al., 2016). How can the users be subliminally induced to focus their attention on the most relevant elements of the narrative? Exploring these challenges in these dynamic environments requires a more complex approach, but also a more ecological realism that sets richer and more relevant solutions for human experience (Zacharov, 2019).

Within this research context and drawing from prior investigation (Salselas, 2020), we aim to explore if synchrony consistency across audiovisual sensory modalities might be a distinctive feature that contributes to make an event stand out from its context. Could auditory and visual information redundancy strengthen the sensory signal in a disruptive, uncorrelated sensory environment? Specifically, could audiovisual synchrony, when visual events are associated with visible trigger movements be salient attentional events? Driven by this enquiry, we test if salient auditory and visual stimuli, that are clearly matched temporally (synchrony), represent a salient event that captures and drives attention. To assess this, we propose an ecologically valid stimuli approach, in a controlled laboratory environment, that aims to provide a comprehensive and relevant analysis of the question.

## Methods

### Participants

Nineteen subjects participated in this study (8 women and 11 men, mean age = 30.4 years, std = 7.4 years). All participants reported normal auditory and visual acuity (no subjects with hearing impairments or severe eye impairment were recruited for this study). All subjects gave informed consent to participate in this study. No invasive or harmless procedures were used. Collected personal data were processed following the General Data Protection Regulation (EU) 2016/679 (GDPR). The experiments were conducted in accordance with the principles stated in the 1964 Declaration of Helsinki and posterior amendments.

### Stimuli

For the stimuli, we have produced four videos with 20 s duration each. The videos capture four scenes performed by actors on a set and were all recorded in the same room, with identical framing (image and sound), using a video camera and an omnidirectional microphone mounted on a (static) tripod. Scenography elements in the four scenes are the same (e.g., set props, actors, clothing, lighting, etc.). The scenes differ exclusively on the actors’ performed actions and their locations within the set, as summarized in Table 1. These audiovisual pieces were produced as to constitute contrasting content relative to *Points of synchronization. Point of synchronization,* or *synch point*, a term coined by Chion, refers to “a salient moment of an audiovisual sequence during which a sound event and a visual event meet in synchrony” (Chion, 2019, p. 58). This presupposes that a visual event is associated with a visible trigger movement and that, additionally, it is synchronous with its corresponding sound (i.e., a synchronous audiovisual transient event) (Bruns & Getzmann 2008).

**Table 1.** Audiovisual stimuli description. While the four scenes have the same setting, they show four different actions. Audio and visual framing is the same for all scenes. Audio was recorded with a mono omnidirectional microphone set at the same location as the video camera. The set is built inside a low reverberance treated room at CARA studios—Estudio 2, Matosinhos, Portugal.

**Scene S1** A chime being blown by a fan.	**Synch Point** Audiovisual events where sounds are associated with respective synchronous visible trigger actions or movements. The chime movement is visibly synchronous with the tinkling sounds it produces.	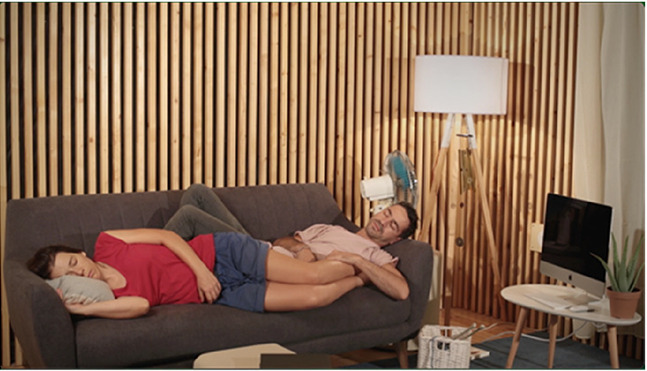
**Scene S2** A woman writing with a pencil in a notebook.	**Synch Point** Hand and pencil movements are visibly synchronous with the writing on paper sound.	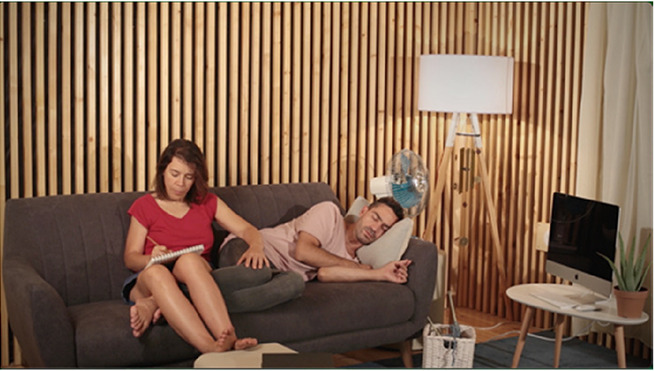
**Scene S3** A woman looking out the window.	**No Synch Point** Visible trigger actions or movements are **not** associated with synchronous sounds. The sound coming from outside does not appear synchronous with any visual element.	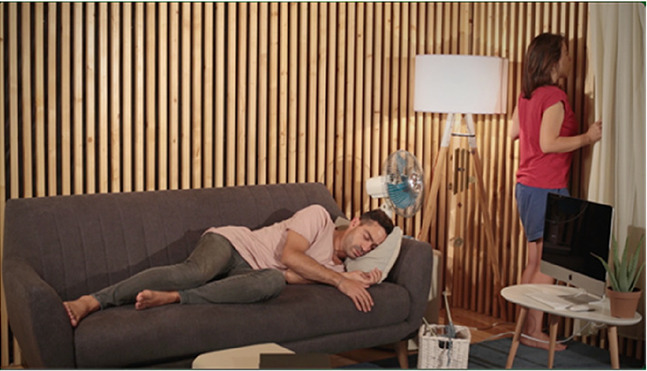
**Scene S4** A man playing a gamepad.	**No Synch Point** Gamepad sounds do not appear synchronous to any visible action.	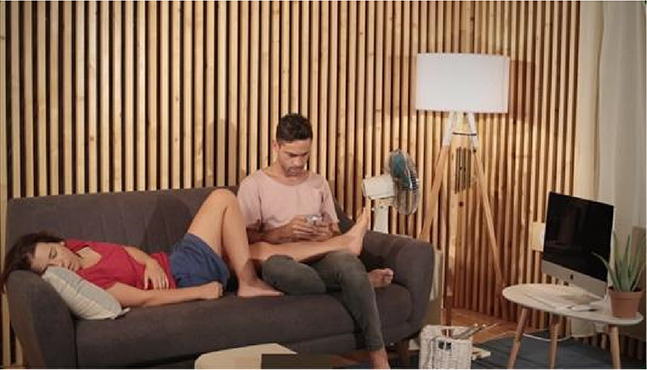

Of the four videos comprising the stimuli for this experiment, two contain synch points, moments where actions—visible trigger events—are associated with synchronous sounds (scenes of a woman writing with a pencil in a notebook and a chime being blown by a fan). Whereas the other two videos do not contain any synch points, that is, in these, the actions—visible events—are not associated with synchronous sounds (scenes of a woman looking out the window accompanied by the muffled sounds coming from the outside and a man playing a gamepad).

The selection of these video scenes resulted from a prior pilot study where the stimuli were tested for possible bias. As a consequence of that, a video containing a set with the two actors watching TV was discarded from the experimental stimuli since a strong visual bias was observed. Additionally, all the produced videos^1^ were controlled for any possible visual bias (see Procedure).

In order to assess orienting attention in terms of participant preference for one video over the other, videos were presented in pairs (simultaneously, on two screens), ensuring that all possible pair combinations were shown to each participant. The generated experimental stimuli were grouped in two sets of video sequences, differing on the combination rules:

- **Set 1** contains all the possible pair combinations of the four videos considering lateral position of presentation (i.e., left side and right side). In this set all videos are muted, thus no sound is considered for the combinations. Consequently, this set consists of 12 video pairs, with a total duration of 4 min. Video pairs were presented sequentially. For each trial and for each participant, a new random order was computed.
- **Set 2** comprises all the possible pair combinations of the four videos considering not only lateral position of presentation (i.e., left side and right side), but also sound-video correspondence. That is, for each pair, only the sound recording of one of the videos is reproduced, and it is taken into account if it corresponds to the video displayed on the left or to the video displayed on the right. As such, in this set, for each two videos (no repetition) there are four possible combinations, resulting in 24 possible video pairings, with a total duration of 8 min. Once again, video pairs were presented sequentially and for each trial and for each participant, a new random order was computed.

For all stimuli presentation, audio was reproduced with 44.1 kHz sample rate at a pre-adjusted comfortable listening level (65 dBa). *VLC* media player (*VideoLan*) was used for playback.

### Procedure

Participants were welcomed in the experimenting booth and instructed about the experimental procedure, where their task would be to observe and, with their gaze, freely explore a sequence of audiovisual content presented in a setup comprising two side-by-side screens and an in-between centered loudspeaker. After this brief introduction, informed consent and basic demographic data was collected.

**Figure 1.**
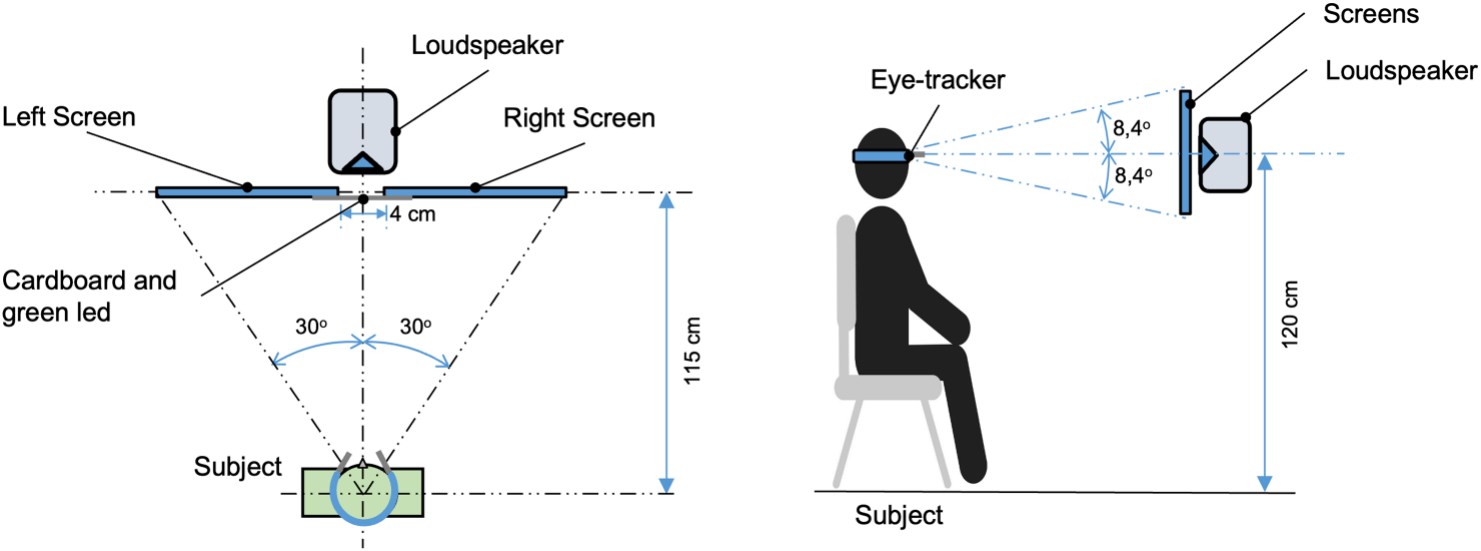
Experimental setup scheme, plan view and side view (not to scale).

Next, participants were guided into a booth and seated on a chair facing two screens (*Asus VG278 LCD 1920 × 1080 monitors*, 27′’) and a loudspeaker (*JBL LSR305*). To ensure distance consistency, a non-swivel fixed position chair was used, height adjustments were made so that participant eyes were at about 120 cm from the floor, such that the center of the screens were at eye-level, with a perpendicular distance of 115 cm (Figure 1).

Subsequently to the adjustments, the eye tracker equipment was mounted and a calibration procedure was conducted for each participant. The eye tracker used was an *Ergoneers Dikablis Professional*. The device has two cameras facing the eyes (one for each eye), which records the position of the pupil and a forward facing scene camera, with a resolution of 1920 × 1080. It was connected to a computer running the D-Lab 3.0 software. This software controlled the eye-tracker, allowing calibration, planning of experiments, and data collection. Calibration was done by running a specific software procedure. The participant was asked to look at four points on its visual scene (one in each quadrant of scene), in sequence, while the same points were marked by the experimenter on a view of the scene camera displayed on the software. This allowed the software to match between the two pupils’ positions (in the eyes’ cameras) and the gaze in the forward-facing camera. The position of the gaze was given in relation to a specific QR marker placed between the screens, with a sampling frequency of 60 Hz.

For the experimental task, stimuli were presented to participants as a sequence of video pairs (see Stimuli), both displayed simultaneously, one on each screen. Meanwhile, participants explored the shown videos while gaze data was recorded by the eye-tracker system. In between pairs of stimuli, at eye level, a green LED lighted up. Participants were asked to fix their gaze on the LED every time it lit up. This ensures that when a new pair of videos appeared, the participant was not already looking towards one of the screens.

The experimental task was organized into two parts. The first part consisted of the presentation of Set 1 (see Stimuli). This video pair sequence had no sound, establishing a control condition with the purpose of detecting any possible bias for video visual preferences.

In the second part, Set 2, an eight-minute-long sequence of video pairs was presented to the participants. The purpose of this part was to verify if participants revealed any preference for the audio-matching videos and, if synchrony had any significant effect on these preferences.

Once the participants had been exposed to all 36 trials (12 in the first part and 24 in the second) the experimental procedure was completed.

Preferences were reflected on the proportion of looking/gaze time between the two screens (defined as areas of interest (AOI)). Gaze location and time data was collected by the eye-tracker and saved on a hard drive for subsequent analysis.

### Data Analysis

Preliminary analysis included the evaluation of eye-tracking system data quality (ETq), determined, for each participant, as the proportion of valid frames (i.e., the ratio of number of frames in which the system was able to determine a set of valid gaze coordinates to total number of frames). This is given by the formula:

(1) $$ETq_{i}=mean\left(\frac{validframes_{1}}{totalframes_{1}},\ldots ,\;\frac{validframes_{n}}{totalframes_{n}}\;,\ldots ,\frac{validframes_{36}}{totalframes_{36}}\right)$$

where:

*i* = from 1 to 19 (participant id);

*n* = from 1 to 36 (trial id);

The determination of the attention behavior is based on the definition of AOIs in the recordings made by the eye-tracker. Two AOIs were set, one for each screen. Participant attention was assumed as directed to one of the screens when gaze coordinates fell within one of the two AOIs. We, thus, determined, for each trial, the proportion of attention frames (gazes within the right or left screens AOIs), as a fraction of the valid frames. We then computed an attention quality metric per participant (ATq), as the mean of proportions of attention frames (Equation 2):

(2) $$ATq_{i}=mean\left(\frac{gazeL_{1+}gazeR_{1}}{validframes_{1}},\ldots ,\;\frac{gazeL_{n+}gazeR_{n}}{validframes_{n}}\;,\ldots ,\frac{gazeL_{36+}gazeR_{36}}{validframes_{36}}\right)$$

With:

*i* = from 1 to 19 (participant id)

*n* = from 1 to 36 (trial id);

Bias in the attention towards images with and without sound was determined based on two indicators: (1) bias in absence of sound and (2) sound induced attention bias.

#### Bias in Absence of Sound

Bias in the attention towards any particular video scene, in the absence of sound, was calculated by determining for each participant and each of the six possible pairings of scenes, the proportion of gazing time towards one particular reference scene (Equation 3).

Considering the pairing of generic scenes SA and SB and their displayed positions, there are the cases of SA,left × SB,right and SB,left × SA,right. Taking SA as the reference scene, we have:

(3) $$Proportion_{ik,AB}=mean\left(\frac{gazeSA_{i,SA\times SB}}{gazeSA_{i,SA\times SB}+gazeSB_{i,SA\times SB}},\frac{gazeSA_{i,SB\times SA}}{gazeSA_{i,SB\times SA}+gazeSB_{i,SB\times SA}}\right)$$

With:

*i* = from 1 to 19 (participant id);

*k* = from 1 to 6 (comparison id);

*A* = 1, 2, or 4; *B* = 2, 3, or 4; and *A* ≠ __*B*;

#### Sound-Induced Attention Bias

To determine how much the attention of a participant is biased towards a scene when the reproduced sound matches that same scene, we computed a metric which we call Auditory-induced Attention Bias (AAB). This is calculated by comparing gazing time of a participant towards a scene when the reproduced sound matches that same scene (pairings in Set 2), compared to when both scenes are muted (pairings in Set 1). The use of the muted conditions as offset aims to correct for individual preferences towards any particular scene. AAB is calculated per participant, for each scene of a (generic) pairing $S_{x}$ versus $S_{y}$ , following the formula:

$$AAB_{x}=P_{x}-P_{x:muted}$$

$$AAB_{y}=P_{y}-P_{y:muted}$$

where $P_{x}$ is the proportion of frames gazing towards *Sx* in the trials with sound matching Sy , $P_{y}$ is the proportion of frames gazing towards *Sy* in the trials with sound matching Sy and, $P_{x:muted}$ and $P_{y:muted}$ , correspond to the proportion of frames gazing towards *Sx* and *Sy* respectively, in the trials without sound.

Comparison of the indicators against reference values was conducted using *t*-tests with *p* values adjusted for multiple comparisons through Bonferroni corrections. A two-way repeated measures analysis of variance (ANOVA) was also computed to compare AABs between scenes with (SP) and without (nSP) sync points, for different nSP × SP pairs of scenes.

## Results

### Preliminary Analysis

Our first step was to analyze the eye-tracking system data quality. The mean value of ETq across participants is *M* = 71%, SD = 10%. One participant with ETq below 50% was discarded from subsequent analyses.

The next step was the calculation of ATq for each participant. The mean value across participants is *M* = 92%, SD = 8%, meaning that participants spent the majority of their task time effectively looking at either one or the other AOI.

### Bias Towards any Particular Video Scene

**Figure 2.**
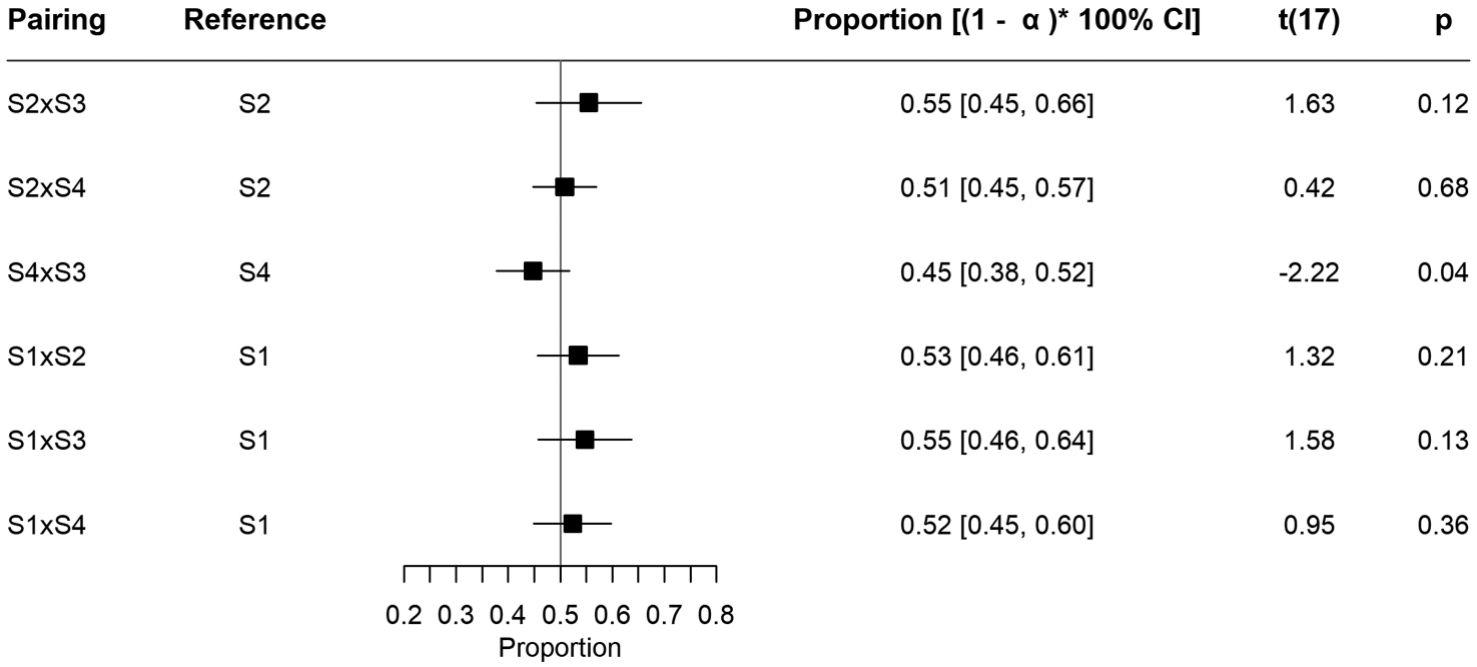
Means of proportion of gazing time towards reference scene.

We proceed to inspect for bias in the attention towards any particular video scene, in the absence of sound. Multiple one sample *t*-tests were performed to test for differences from the 0.5 proportion. As there were six pairs of scenes, α was defined as 0.05/6, following the Bonferroni criteria. Figure 2 shows each scene pairing the mean proportion and the respective (1− α )×100% confidence intervals, as well as the results of the tests. Mean values range between 0.45 and 0.55. Differences to 0.5 were non-significant for all pairs.

### Sound as a Salient Event

**Figure 3.**
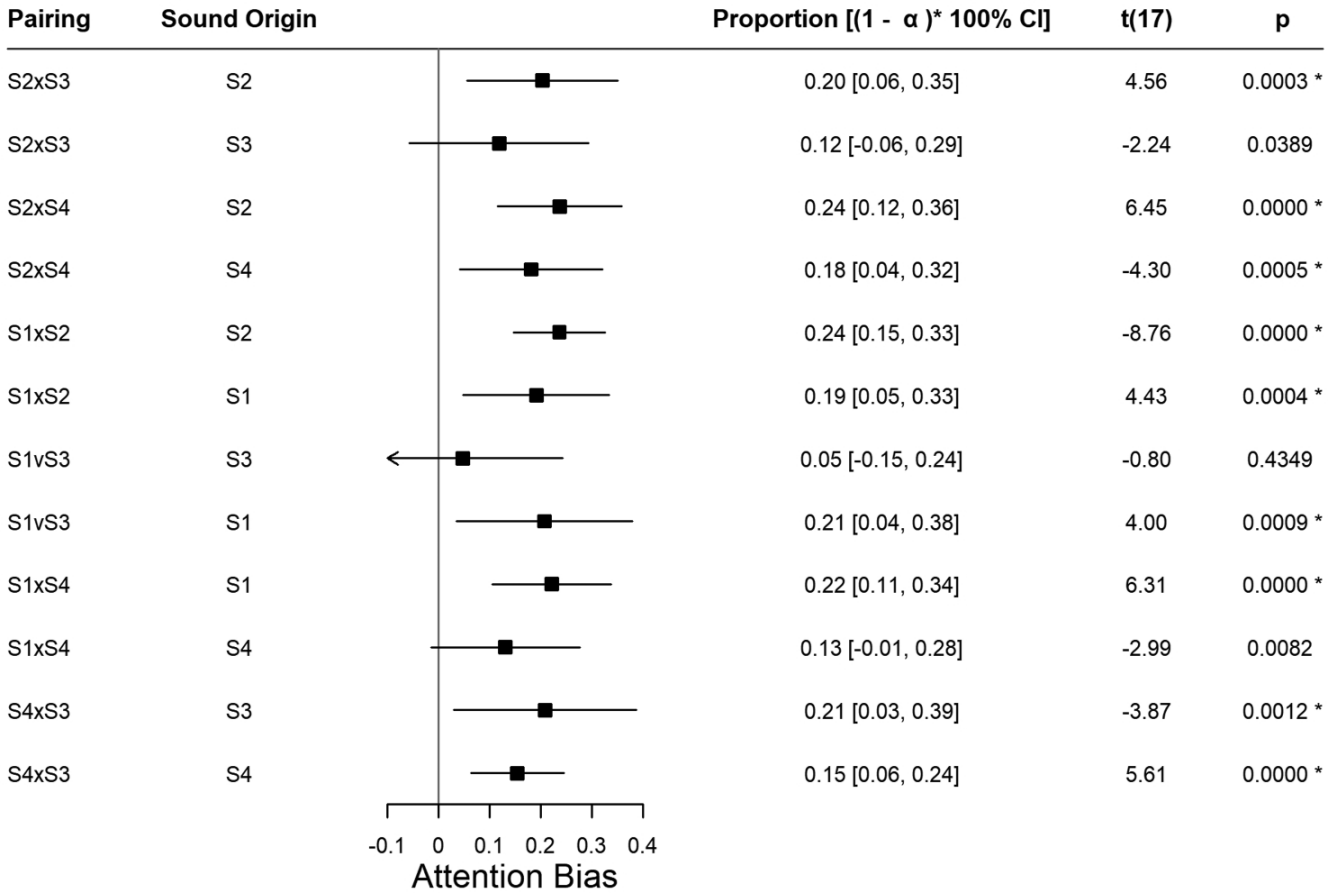
Average and 95% confidence intervals for the mean of AAB*x* and AAB*y*, for each pairing of scenes.

We assessed if having audio matching one particular scene was by itself enough to introduce a bias in attention towards that scene. To do so we calculated AAB for each of the sounds in each of the scene pairs. Multiple *t*-tests were performed to test the differences against 0, with Bonferroni correction α __= 0.05/12. Figure 3 reports the average and (1− α )×100% confidence intervals for the mean of AAB*x* and AAB*y*, for each pairing of scenes, as well as the statistics for the *t*-tests. All biases are different from zero, except S3 when paired with S1 or S2, or S4 when paired with S1. In all the other conditions the listening of sound biased the attention towards the corresponding scene.

**Figure 4.**
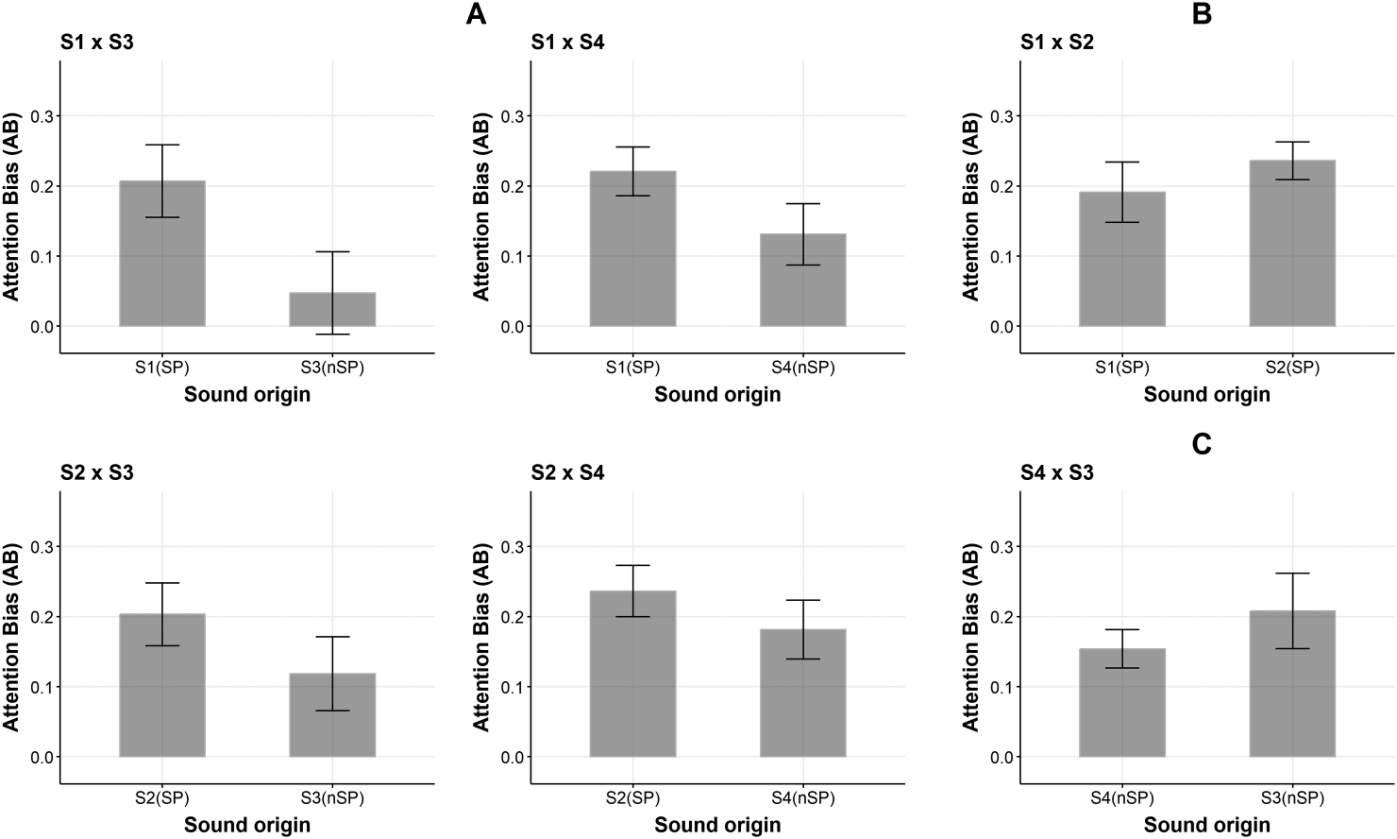
Proportions of gazing time for each scene pairing condition. (A) Pairings of *SP vs nSP*; (B) Pairing of *S1(SP) vs S2(SP)*; (C) Pairing of *S4(nSP) vs S3(SP)*.

Lastly, we wanted to determine if the AAB induced by the sounds is greater towards a scene containing Synch Points when paired with another without Synch Points. To do so we analyzed the trials in which the scene pair included a scene with Synch Points (SP) and a scene with no Synch Points (nSP), namely: *S1(SP) vs S3(nSP), S1(SP) vs S4(nSP), S2(SP) vs S3(nSP) and S2(SP) vs S4(nSP);* (Figure 4A).

We conducted a **two-way repeated-measures ANOVA** to examine AAB. As factors we considered Scene Pairing (four levels) and the Sound Origin (two levels, including *sound matching the scene with Synch Points*—SP, s*ound matching the scene with no Synch Points*—nSP). Results show a significant effect of Sound Origin, F(1, 17) = 51.37, *p* < .001, η^2^ = 0.751 and no effect of Scene Pairing, F(3, 51) = 0.41, *p* = .745. Thus, matching sounds result in greater AABs when the corresponding scene has sync points. Interaction between Scene Pairing and Sound Origin was non-significant, F (3, 51) = 0.09, *p* = .97. Additionally, a statistical power analysis was carried out to infer the probability of rejecting the null hypothesis (Sound Origin (Synch Point or no Synch Point) does not have an effect on AAB). The analysis makes use of SuperPower package (Lakens & Caldwell, 2021), and assumes for the two-way within (2w*4w) repeated measures design, a correlation factor *r = *.5, alpha level *p < .*001 and a sample population *n = *18. The results of the analysis reveal that power is higher for the main effect of Sound Origin (>90%), and, as expected, very low for the effect of Scene Pairing and interaction between Sound Origin and Scene pairing.

For the sake of completion, we conducted paired *t*-tests to analyze the bias towards Sound Origin when pairing two videos with sync points: *S1(SP) vs S2(SP)*—Figure 4B and two videos with no sync points: *S3(nSP) vs S4(nSP)*—Figure 4C. No significant differences were found for *S1 vs S2*, *t*(17) = −0.91, *p* = .374 nor for *S3 vs S4*, *t*(17) = −0.96, *p* = .349.

Overall our results show that sound from any particular scene induced an attention bias towards that scene. Most importantly, it also showed that the presence of Synch Points in a scene significantly increases the attention bias towards that scene.

## Discussion

In this research work, we have examined if consistency across audiovisual sensory modalities, in terms of synchrony, could be a distinctive feature contributing to an event to stand out from its context (saliency). Particularly, we have examined the role of Synch Points—a visible trigger event that is synchronous with its corresponding sound. For this examination, we designed and conducted an experimental study testing the hypothesis that synchronous auditory and visual stimuli represent a salient event that captures and drives attention.

The experimental study was designed so that *attention*, that is, the process of focal selection of information or subsets of information, could be assessed. Consequently, for the experimental task, participants had to observe a set of paired video scenes displayed simultaneously, on two screens, while using an eye-tracking device. The eye-tracking kit enabled recording data relative to where participants were fixating their gazes at, thus, identifying their attentional focus between the two screens. The cases where only one scene from the displayed pair matched the reproduced audio, allowed to analyze if participants’ preferences, measured in terms of gaze time, were due to synchronous audiovisual stimuli, particularly, to Synch Points (see Stimuli for detailed description).

In the first part of the experimental task, where stimuli were exclusively visual, only the paired video images were presented (no sound), we could assure that there was no pattern of bias towards any particular video image. This control was thoroughly conducted since, in a previous pilot stage of this experimental study, a video scene was discarded as it elicited a pattern of visual bias relative to all other video scenes.

Indeed, results showed that no particular video scene consistently triggered any visual preference in relation to its counterpart paired scene. Scene *S3* exhibited a subtle visual saliency when displayed along with scene *S4*. We hypothesize that this may be due to the fact that in *S3* one actor is standing by the window, contrasting with the other scenes. This relatively different spatial location of the actress might have caused some curiosity and expectation to what action might occur next. However, this preference is not consistent throughout the alternative pairing scenes (i.e., scene *S1* and scene *S2*). Consequently, we consider that the results from this first part of the task validate the video images stimuli as neutral. Additionally, it is plausible to suggest that for the subsequent stages of the experimental task, the novelty effect of the scenes’ imagery becomes minimal. Then, sound becomes the significant factor that might drive attention towards a particular location, or enhance the detection of a salient event, that is, prompting attentional bias and orienting preferences.

Following this track, we proceeded with the analysis, assessing if matching sound with one particular scene of a pair, could induce any preference and thus, attentional bias. A metric was proposed, *Auditory-induced Attention Bias* (see Results). This metric provided a means to assess sound as the biasing factor, establishing, this way, a measurement parameter. Indeed, the results show that sound causes an attentional bias towards the particular scene it matches. These results agree with previous research (Iordanescu et al., 2008; Noesselt et al., 2008; van Eijk et al., 2008).

A closer examination of the results reveals that this bias stands out in scenes *S1* and *S2*. These two scenes differ from Scenes *S3* and *S4* as having been designed to integrate Synch Points. Synch Points, previously described as visible trigger events that are associated with synchronous sounds, enabled us to isolate synchrony as the attentional bias factor, distinguishable from the condition of solely matching sound, as in scenes *S3* and *S4*.

Consequently, the results show that consistency across audiovisual sensory modalities is revealed to be a distinctive feature. However, when combined with synchrony, it clearly emphasizes the biasing, causing a trigger for orienting, that is, focal attention towards the particular scene that contains the Synch Point. Thus, as hypothesized, synchrony revealed to stand out from “matching sound” as a saliency factor, contributing to the strengthening of the focal attention.

For the experimental protocol, we have carefully assured that good and rigorous experimental practices were implemented, including a number of control conditions for any possible disturbing variables and bias. These control conditions enabled us to mitigate noisy variables’ interference. Moreover, the eye-tracking equipment, combined with the *Auditory-induced Attention Bias* metric, provided a reliable means to study attention and assess orienting more objectively, which can be further explored and used in future research.

However, we are aware that the ecological nature of the stimuli (i.e., the video scenes) is complex, contrasting with simple laboratory stimuli such as *beeps and flashes*, commonly used in synchrony experimental studies (e.g., Noesselt et al., 2008; van Eijk et al., 2008). Conversely, this ecological experimental design approach pretends to be closer to the ordinary life situation, which provides a more relevant and meaningful assessment of the problem (Matusz et al., 2019; Zacharov, 2019). Particularly, when considering new challenges arising from emergent technologies, such as immersive audiovisual environments, adopting an experimental approach that is much closer to the users’ conditions, may pave the way to a greater understanding of human interaction with these technologies. As such, the reported results may be of relevance for the context of immersive non-linear media and may contribute to new sound design solutions for capturing users’ attention in a subliminal approach, alternatively to commonly used warning sounds such as artificial tones which can be disruptive to the immersive experience (Jennett et al., 2008).

## Notes

1. The videos can be found in audiovisual_attention_videos.
